# Sugar-fermenting yeast as an organic source of carbon dioxide to attract the malaria mosquito *Anopheles gambiae s.s.*

**DOI:** 10.1186/1475-2875-9-S2-O29

**Published:** 2010-10-20

**Authors:** Renate C Smallegange, Wolfgang H Schmied, Karel J van Roey, Niels O Verhulst, Jeroen Spitzen, Wolfgang R Mukabana, Willem Takken

**Affiliations:** 1 Laboratory of Entomology, Wageningen University, P.O. Box 8031, 6700 EH, Wageningen, The Netherlands; 2 International Centre of Insect Physiology and Ecology, P.O.Box 30772 - 00100, GPO, Nairobi, Kenya; 3 School of Biological Sciences, University of Nairobi, P.O. Box 30197 - 00100 GPO, Nairobi, Kenya

## Background

Carbon dioxide (CO_2_) plays an important role in the host-seeking process of opportunistic, zoophilic and anthropophilic mosquito species and is therefore commonly added to mosquito sampling tools. The African malaria vector *Anopheles gambiae* Giles *sensu stricto* is attracted to human volatiles augmented by CO_2_. We investigated whether CO_2_, usually supplied from gas cylinders acquired from commercial industry, could be replaced by CO_2_ derived from fermenting yeast (yeast-produced CO_2_).

## Methods

Trapping experiments were conducted in the laboratory, semi-field and field, with *An. gambiae s.s.* as the target species. MM-X traps were baited with volatiles produced by yeast-sugar solutions, prepared in bottles. Catches were compared with traps baited with industrial CO_2_. The additional effect of human odours was also examined.

## Results

Traps baited with yeast-produced CO_2_ caught significantly more mosquitoes than unbaited traps and also significantly more than traps baited with industrial CO_2_, both in the laboratory and semi-field. Adding yeast-produced CO_2_ to traps baited with human odour significantly increased trap catches. During the field trials, traps baited with yeast-produced CO_2_ caught similar numbers of *An. arabiensis* Patton as traps baited with industrial CO_2_. Addition of human odour increased trap catches.

## Conclusions

We conclude that yeast-produced CO_2_ can effectively replace industrial CO_2_ for sampling of *An. gambiae s.s..* This will significantly reduce costs and allow sustainable mass application of odour-baited devices for mosquito sampling in remote areas.

